# Raising root zone temperature improves plant productivity and metabolites in hydroponic lettuce production

**DOI:** 10.3389/fpls.2024.1352331

**Published:** 2024-04-16

**Authors:** Sota Hayashi, Christopher P. Levine, Wakabayashi Yu, Mayumi Usui, Atsuyuki Yukawa, Yoshihiro Ohmori, Miyako Kusano, Makoto Kobayashi, Tomoko Nishizawa, Ikusaburo Kurimoto, Saneyuki Kawabata, Wataru Yamori

**Affiliations:** ^1^ Institute for Sustainable Agro-ecosystem Services, The University of Tokyo, Nishitokyo, Tokyo, Japan; ^2^ Plants Laboratory Inc., Nishitokyo, Japan; ^3^ Faculty of Life and Environmental Sciences, University of Tsukuba, Tsukuba, Japan; ^4^ Tsukuba-Plant Innovation Research Center (T-PIRC), University of Tsukuba, Tsukuba, Japan; ^5^ Riken Center for Sustainable Resource Science, Yokohama, Kanagawa, Japan; ^6^ National Institute of Technology, Kisarazu College, Kisarazu, Chiba, Japan

**Keywords:** root zone temperature, metabolome, pigment, ionome, lettuce (Lactuca sativa), plant factory, hydroponics, nutrient film technique

## Abstract

While it is commonly understood that air temperature can greatly affect the process of photosynthesis and the growth of higher plants, the impact of root zone temperature (RZT) on plant growth, metabolism, essential elements, as well as key metabolites like chlorophyll and carotenoids, remains an area that necessitates extensive research. Therefore, this study aimed to investigate the impact of raising the RZT on the growth, metabolites, elements, and proteins of red leaf lettuce. Lettuce was hydroponically grown in a plant factory with artificial light at four different air temperatures (17, 22, 27, and 30°C) and two treatments with different RZTs. The RZT was raised 3°C above the air temperature in one group, while it was not in the other group. Increasing the RZT 3°C above the air temperature improved plant growth and metabolites, including carotenoids, ascorbic acids, and chlorophyll, in all four air temperature treatments. Moreover, raising the RZT increased Mg, K, Fe, Cu, Se, Rb, amino acids, and total soluble proteins in the leaf tissue at all four air temperatures. These results showed that raising the RZT by 3°C improved plant productivity and the metabolites of the hydroponic lettuce by enhancing nutrient uptake and activating the metabolism in the roots at all four air temperatures. Overall, this research demonstrates that plant growth and metabolites can be improved simultaneously with an increased RZT relative to air temperature. This study serves as a foundation for future research on optimizing RZT in relation to air temperature. Further recommended studies include investigating the differential effects of multiple RZT variations relative to air temperature for increased optimization, examining the effects of RZT during nighttime versus daytime, and exploring the impact of stem heating. This research has the potential to make a valuable contribution to the ongoing growth and progress of the plant factory industry and fundamental advancements in root zone physiology. Overall, this research demonstrates that plant growth and metabolites can be improved simultaneously with an increased RZT relative to air temperature. This study serves as a foundation for future research on optimizing RZT in relation to air temperature. Further recommended studies include investigating the differential effects of multiple RZT variations relative to air temperature for increased optimization, examining the effects of RZT during nighttime versus daytime, and exploring the impact of stem heating. This research has the potential to make a valuable contribution to the ongoing growth and progress of the plant factory industry and fundamental advancements in root zone physiology.

## Introduction

Plant factories with artificial light are increasingly popular for the hydroponic cultivation of leafy greens. Compared to conventional outdoor field operations, plant factories provide exceptional control over environmental conditions (Graamans et al., 2018). Environmental management in plant factories covers a wide range of abiotic parameters, such as temperature, relative humidity, vapor pressure deficit, carbon dioxide concentration, light intensity, light quality, and culture mediums. All of these abiotic parameters, when optimized, enable efficient and stable production of high-quality crops regardless of the season or weather conditions (Morrow, 2008; Takatsuji, 2010; Merrill et al., 2016).

A completely abiotic, stress-free environment is not necessarily optimal for producing leafy greens for human consumption. Specific abiotic stressors that amplify stress responses, such as root zone temperature (RZT) and light quality in plant factories, can add economic value to plants (Dresselhaus and Hückelhoven, 2018). Previous studies have shown that RZT can influence various root physiological processes, such as water and nutrient uptake, photosynthesis, and assimilate distribution (Udagawa et al., 1991; Klock et al., 1997; Yamori et al., 2022; Levine et al., 2023). Root growth increased linearly with increasing RZT from a minimum to an optimum temperature (Arkin and Taylor, 1981), although further increases in RZT were accompanied by a rapid decrease in root and shoot growth (Arkin and Taylor, 1981). Furthermore, during cool seasons, heating RZT by electric heating cables increased lettuce shoot weight (Bumgarner et al., 2012). Also, research has been focused in plant factories to enhance the biosynthesis of some secondary metabolites, which include total chlorophyll, total carotenoids, ascorbic acid, anthocyanin (Kong and Nemali, 2021; Levine et al., 2023; Van De Velde et al., 2023). It has been indicated that the regulation of RZT increased leaf nutrient elements (Moccio et al., 2024), and that low RZT treatments increased anthocyanin concentrations (Islam et al., 2019).

Based on the previous studies, we hypothesized that increasing the RZT a few degrees higher than the air temperature may improve the growth and quality of plants. Recent experiments demonstrating that the optimal RZT depends on the air temperature (Yamori et al., 2022; Levine et al., 2023) led us to investigate raising the RZT at various air temperatures. To our knowledge, no studies have investigated the effect of temperature changes of several degrees to the RZT relative to varying air temperatures on plant growth and the metabolites for this lettuce cultivar, although numerous studies have already examined the effects of air temperature on plant growth and showed that low or high air temperatures negatively affect plant physiological processes, such as photosynthesis, respiration, growth, and development, which consequently result in reduced crop yields (Yamori et al., 2005, 2010; Wahid et al., 2007; Theocharis et al., 2012; Hasanuzzaman et al., 2013; Way and Yamori, 2014; Yamori et al., 2014; Qu et al., 2021). Based on these prior studies, we selected a slight 3°C increase in RZT to ensure that we did not cross the threshold or tipping point that could cause a rapid decrease in root and shoot growth.

In considering the effects of raising RZT on plant growth and metabolites, it is important to clarify the physiological processes to understand the environmental response to it better. Ionome analysis has been used to reveal the uptake and translocation of mineral elements in plants (Quadir et al., 2011; Nicolas et al., 2019), while metabolite profiling analysis has been widely used to reveal changes in metabolites in response to various environments, including light intensity, light quality, and UV-B irradiation (Kusano et al., 2011; Goto et al., 2016; Kitazaki et al., 2018). By combining multiple omics analyses, it is possible to gain a more comprehensive understanding of RZT’s impact on plants’ physiological processes. These studies can be highly beneficial and can provide valuable insights into how RZT affects the different aspects of plant growth and development. Overall, the main objective of this study is to analyze the effects of raising RZT on the plant growth, elements, and metabolites of ‘Red Fire’ red leaf lettuce plants grown at different air temperatures. Four air temperature treatments (17, 22, 27, and 30°C) and two RZT treatments (no root zone heating and raising the root zone temperature 3°C above the air temperature) were applied. The effects of raising the RZT by 3°C on lettuce growth and functional components were comprehensively investigated by ionome and metabolite profiling analyses.

## Materials and methods

### Plant materials and treatments

The seeds of ‘Red Fire’ red leaf lettuce (Takii Seed Co., Kyoto, Japan) were used. All the experiments on the red leaf lettuce, including the collection of plant material, were in compliance with relevant institutional, national, and international guidelines and legislation.

Growth conditions were controlled at 62.9 ± 6% relative humidity and 16 hours of photoperiod artificial light using white LED lights (TecoG II-40N2-5-23, Toshin Electric Co., Ltd., Osaka, Japan). During the seed propagation period, the photosynthetic photon flux density (PPFD) was maintained at 120 ± 10 µmol m^−2^ s^−1^ in the daytime. The seeds were sown in seedling trays with a sponge substrate at 20°C air temperature with a 13-day propagation time until they had 2-3 true leaves. After 13 days, the plants were transplanted to where the experiment was conducted and acclimated for 3 days before the 16 day experiment started. The PPFD was then increased to 200 ± 20 µmol m^−2^ s^−1^ using white LED light (TecoG II-40N2-5-23, Toshin Electric Co., Ltd., Osaka, Japan). The plants were grown in a custom-built nutrient film technique (NFT) system and supplied with a nutrient solution, GG liquid A and B stock solutions (Green Co., Ltd, Fukuoka, Japan), with an EC of 1.00 ± 0.05 dS/m. Filtered reverse osmosis water was used at all stages of lettuce growth from the seedling stage. There were eight treatments, four air temperatures (17, 22, 27, and 30 ± 1°C), and two treatments in which the temperature of the nutrient solution was either raised 3°C above the air temperature or not heated. The temperature of the nutrient solution, also called root zone temperature (RZT) was controlled with a heater (NHA-065, Marukan Co., Ltd., Osaka, Japan) to maintain a temperature of 3°C above the respective air temperature treatment. The temperature of the nutrient solution was continuously monitored at the root zone area in the NFT system to ensure the plants received proper experimental treatments throughout the experiment. A total of 72 plants were randomly selected and divided into three treatments. In our experiment, two 12-cell trays with 24 plants were propagated in an NFT system using a 30-liter reservoir. This was considered to be one experimental unit. Our experiment involved three treatments with different RZTs, as described below, and one experimental run, which included one experimental unit (1 NFT system with 24 plants). Data from the experiment were taken 19 days after the plants were transplanted to the NFT system (32 days after the seeds were sown).

### Plant growth

To analyze the shoot and root dry weights of the plants, the shoots and roots from every plant were separated 32 days after seeding, placed in paper envelopes, and then dried at 80°C in a constant-temperature oven for about two weeks. Leaf mass per area (LMA) was also determined by cutting the leaf blade of the largest leaf with a leaf puncher and dividing its dry weight by its leaf area.

### Determination of metabolites

Total chlorophyll A+B, total carotenoids (α-Carotene, β-Carotene, zeaxanthin, violaxanthin, lutein, and neoxanthin), and ascorbic acid concentrations were quantified for the analysis of the metabolites. Two 0.56 cm^2^ holes were punched in the center of the largest leaf blades by a hole puncher, and 1.0 mL of 80% acetone was added to the cut sample and ground with a mortar and pestle to extract chlorophyll and carotenoids. The acetone extract was centrifuged at 12,000 rpm for 5 minutes, and the supernatant was used for the analysis. The analysis was performed by measuring absorbance at 750.0, 636.6, 646.6, and 470.0 nm using a UV-Vis-NIR spectrophotometer (UV-2700, Shimadzu Corporation, Kyoto, Japan), and the concentrations of chlorophyll and carotenoids were determined using equations derived from the previous studies (Lichtenthaler, 1987; Porra et al., 1989). A reflectometer (RQ Flex plus, Merck Darmstadt, Germany) quantified the ascorbic acid in the leaves of plants in each treatment (Yamori et al., 2022).

### Ionome analysis

Plant samples were dried for 3 days in a 70°C oven. The weight of dried samples was set to 40–50 mg in a single biological sample. Each sample was digested with nitric acid (HNO_3_) and hydrogen peroxide (H_2_O_2_) (FUJIFILM Wako Pure Chemical Corporation, Osaka, Japan) as follows: 30 min at 80°C and 1 h at 120°C with 2 ml HNO_3_; 1 h at 120°C after adding 0.5 ml HNO_3_ and 0.5 ml H_2_O_2_; and overnight at 80°C until the samples were completely dried. After digestion, the dried pellets were dissolved in 0.08 M HNO_3_. The elemental concentrations (phosphorus [P], potassium [K], calcium [Ca], magnesium [Mg], sulfur [S], iron [Fe], manganese [Mn], boron [B], zinc [Zn], molybdenum [Mo], copper [Cu], nickel [Ni], sodium [Na], cobalt [Co], lithium [Li], germanium [Ge], arsenic [As], selenium [Se], rubidium [Rb], strontium [Sr], cadmium [Cd] and cesium [Cs]) in the samples were measured by inductively coupled plasma mass spectrometry (ICP-MS) (Agilent 7800, Agilent Technologies Co., Ltd, Japan).

### Metabolite profiling analysis

Metabolite profiling was conducted using gas chromatography-time-of-flight-mass spectrometry (GC-MS), as described by (Kusano et al., 2007), but with slight modifications. Six biological replicates were used for the analysis. Metabolites were extracted from each leaf and root sample at a 2.5 mg dry weight tissue concentration per ml of extraction solution (methanol: chloroform: water = 3:1:1 v/v/v), and the extracted samples were methoxylated then subsequently trimethylsilylated. A sample equivalent to 5.6 µg dry weight of derivatized samples was then subjected to GC-MS, and the data obtained (NetCDF format) were transferred to MATLAB version 2011b (MathWorks, MA, USA) software. We used NIST/EPA/NIH Mass Spectral Library 14 (NIST14), an in-house metabolite library, and Golm Metabolome Database (GMD) for identification according to their RI and comparison with the reference mass spectra in the libraries (Kusano et al., 2007). The chromatograms were preprocessed using the high-throughput data analysis method (Jonsson et al., 2005) and were normalized using the cross-contribution compensating multiple standard normalization algorithm (**Supplementary Figure S1**) (Redestig et al., 2009).

### Determination of protein concentrations

Protein extracted from leaves was quantified by a protein-assay kit (Bradford Plus Protein Assay Kit with Dilution-Free BSA Protein Standards, Cat. No. A55866, Thermo Fisher Scientific, Massachusetts, United States), using the method developed by Bradford (1976). The analysis was carried out at a temperature of 22°C. This was primarily because our protein assay test was executed under room temperature conditions.

### Energy consumption analysis

To determine the energy consumption of raising the RZT by 3°C, the energy consumption of heating the lettuce per 1 gram of fresh shoot biomass was assessed. The amount of electricity in kilowatt-hours (kWh) it took to raise the temperature of 10 liters of water by 3°C per day was measured. Energy consumption was calculated by multiplying the amount of electricity by the volume of water in the hydroponic cultivation system and by the 15 growing days in this system. This was then divided by the number of plants in the growing system. Lastly, the electricity values per plant were divided by the fresh weight shoot biomass in grams of the plant in order to get a quantitative value of energy consumption (kWh) per 1 g fresh weight of lettuce. A wattmeter measured how much electricity was consumed during the water heating process (EC-04, Custom Co., Ltd, Tokyo, Japan). It is important to note that energy requirements differ based on shelf designs, seasons, starting water temperature, and other factors. Based on many changing variables that would go into more comprehensive energy analysis, the calculations in this study may not be well representative of other production systems, and it’s meant to serve as a general example of one particular operation in Japan.

### Statistical analysis

A Tukey-Kramer honest significant difference test at α = 0.05 was performed for the means of measurement values to determine significant differences among the measured parameters. For elemental and metabolite concentrations, significance difference tests of the means compared to the 22°C treatment (the control) were performed with the drc 3.0–1 package (Ritz et al., 2015) of the R 3.6.2 (R Core Team, 2017) software, using the graphical interface RStudio Desktop 1.1.4.6.3 (RStudio Team, 2015). The principal component analysis was also conducted using R-Studio (v.4.0.3). Metabolic profile data were analyzed using weighted correlation network analysis (WGCNA) (DiLeo et al., 2011). The Kyoto Encyclopedia of Genes and Genomes was used to search for metabolite pathways. MetaboAnalyst 4.0 software was used for pathway analysis and visualization (Liu et al., 2015). These WGCNA elements and metabolites were classified into two modules (Module1 and Module2), based on their correlation with shoot and root dry weights, chlorophyll, ascorbic acid, and the soluble proteins of leaves and roots. The experiment was repeated three times to ensure the results remained consistent.

## Results

### Effect of raising RZT on plant growth and metabolites

**Figure 1:**
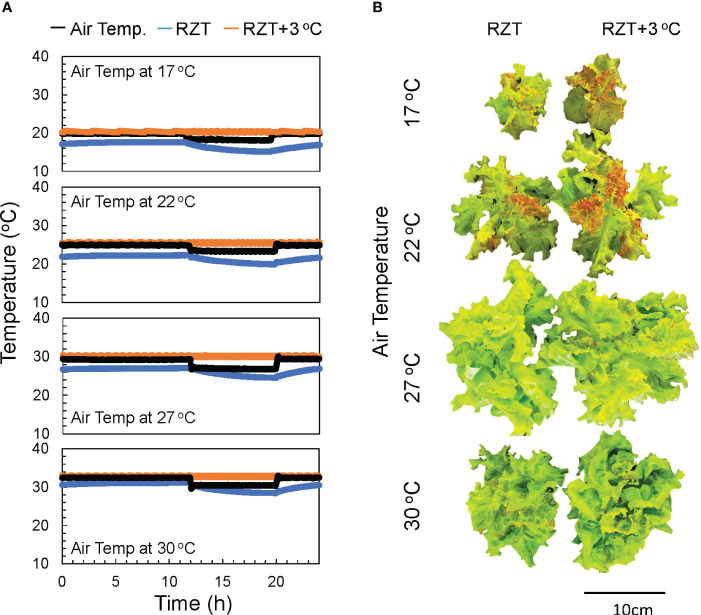
The air temperatures in the 17, 22, 27, and 30°C treatments and the water temperatures over time in the heated (RZT+3 °C) and non-heated treatments (RZT) are shown in **(A)**. The top view of ‘Red Fire’ red leaf lettuce from each treatment harvested 32 days after sowing **(B)** is shown.

The plants in this study were grown under eight treatments, four air temperatures (17, 22, 27, and 30°C), and two treatments with different RZTs. In one group, the RZT was raised 3°C above the air temperature (RZT+3°C), and in the other group, it was not (RZT) (**Figure 1A**).

**Figure 2:**
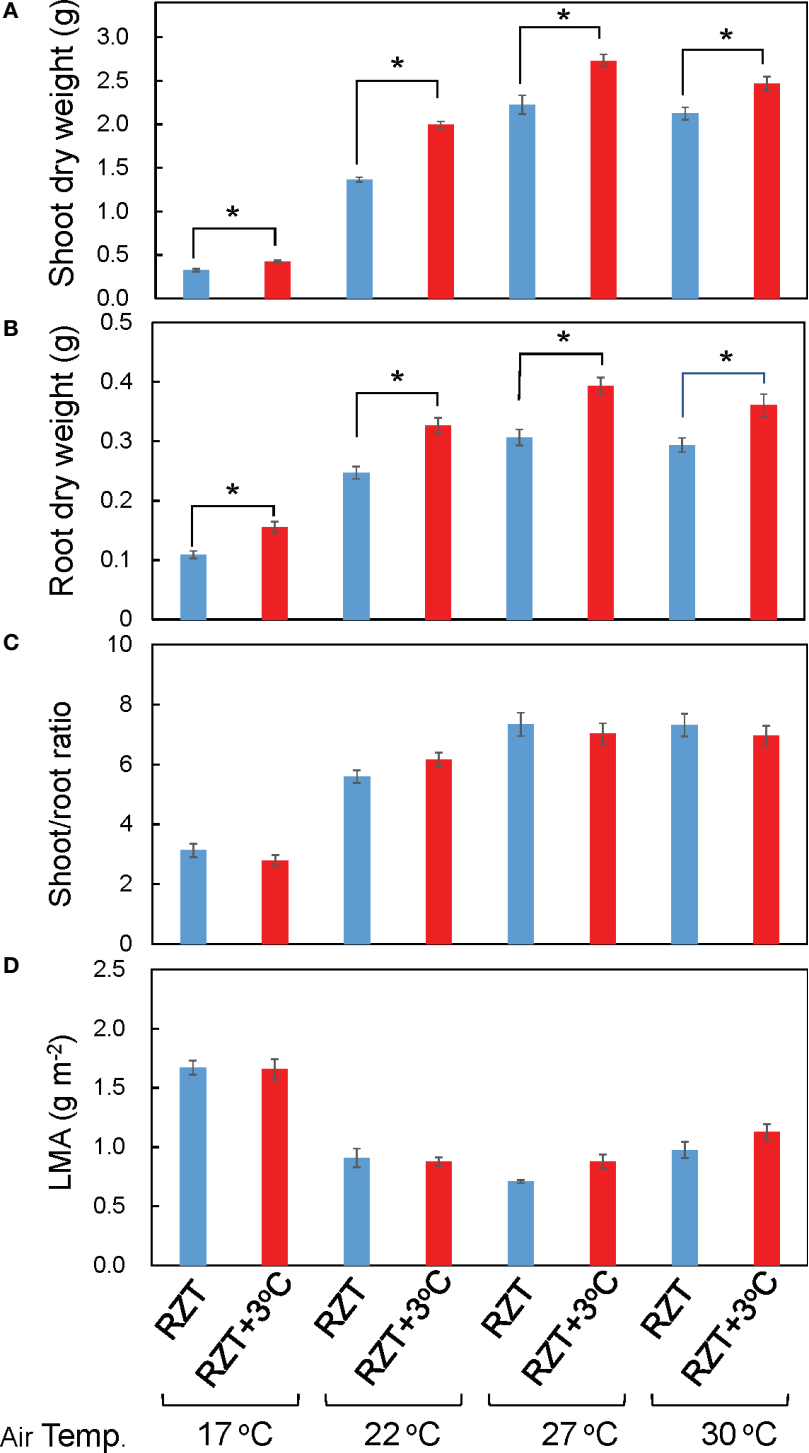
Shoot dry weight **(A)**, root dry weight **(B)**, shoot/root ratio **(C)**, and LMA **(D)** of ‘Red Fire’ red leaf lettuce grown under all the different treatments are shown. The bars are standard errors (n = 6-9). The mark * indicates significant differences between treatments at the same temperature by Tukey’s HSD test (5% level of significance).

**Figure 3:**
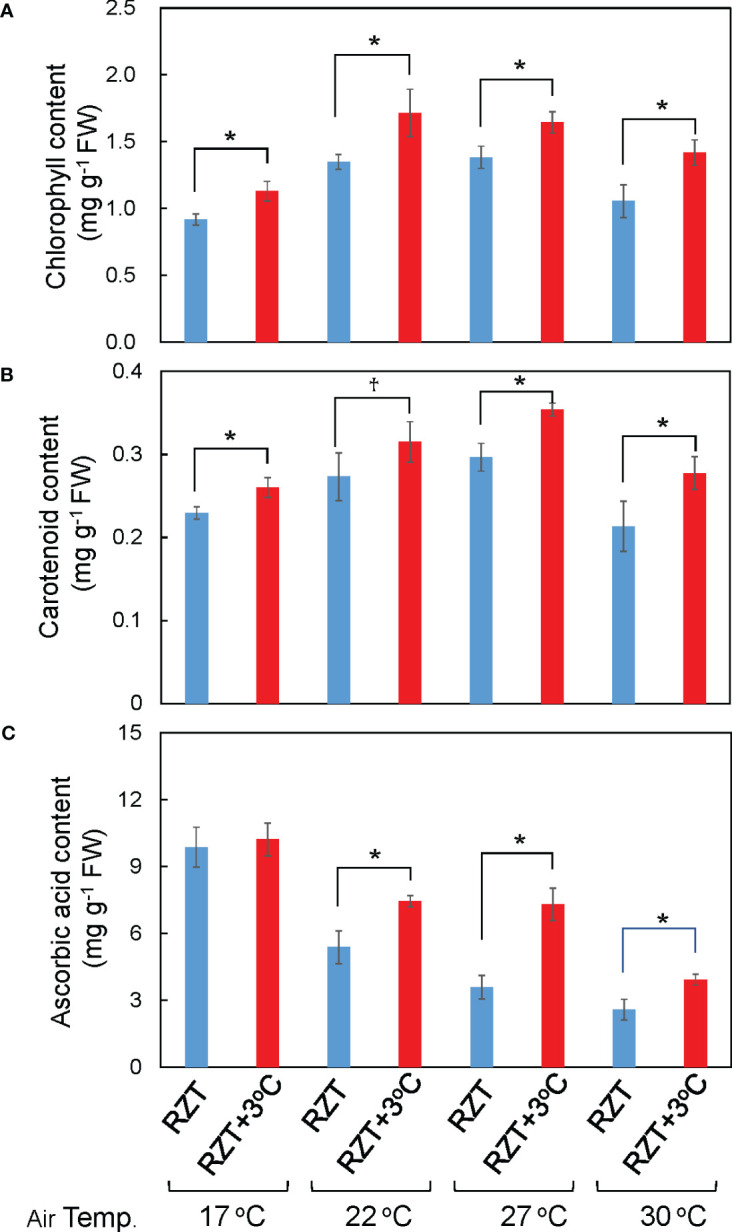
The total chlorophyll **(A)**, total carotenoid **(B)**, and ascorbic acid concentrations **(C)** of ‘Red Fire’ red leaf lettuce grown in different air and RZT treatments are shown. Bars are standard errors (n = 4-7). Statistical differences between treatments at the same temperature by Tukey’s HSD test (5% level of significance). * (significant level, 5%) and ^†^ (marginal significant level, 10%).

Shoot and root dry weights were significantly increased by raising the RZT by 3°C at all air temperature treatments (**Figures 1B**, **2A, B**). The 17°C air temperature treatment with the 3°C raised RZT had 23% greater shoot dry mass and 30% greater root dry mass relative to its corresponding unheated treatment (**Figures 2A, B**). The 22°C air temperature treatment with the 3°C raised RZT had 31% greater shoot dry mass and 24% greater root dry mass relative to its corresponding unheated treatment (**Figures 2A, B**). The 27°C air temperature treatment with the 3°C raised RZT had 18% greater shoot dry mass and 22% greater root dry mass relative to its corresponding unheated treatment (**Figures 2A, B**). Lastly, the 30°C air temperature treatment with the 3°C raised RZT had 14% greater shoot dry mass and 19% greater root dry mass relative to its corresponding unheated treatment (**Figures 2A, B**). The maximum dry weight of shoots and roots was observed with RZT+3°C at 27°C (**Figures 2A, B**). However, the shoot/root ratio and LMA were not significantly changed by raising the RZT at any of the four air temperatures (**Figures 2C, D**). All root zone heating treatments increased carotenoid and chlorophyll contents relative to their corresponding nonheated treatments under all four air temperatures (**Figures 3A, B**). Also, ascorbic acid increased at all air temperatures except 17°C (**Figure 3C**). The 17°C air temperature treatment with the 3°C raised RZT had 19% greater chlorophyll content and 12% greater carotenoid content than its corresponding unheated treatment (**Figures 3A, B**). The 22°C air temperature treatment with the 3°C raised RZT had 21% greater chlorophyll content, 13% greater carotenoid content, and 28% greater ascorbic acid content relative to its corresponding unheated treatment (**Figures 3A-C**). The 27°C air temperature treatment with the 3°C raised RZT had 16% greater chlorophyll content, 16% greater carotenoid content, and 51% greater ascorbic acid content relative to its corresponding unheated treatment (**Figures 3A-C**). Lastly, the 30°C air temperature treatment with the 3°C raised RZT had 26% greater chlorophyll content, 23% greater carotenoid content, and 34% greater ascorbic acid content relative to its corresponding unheated treatment (**Figures 3A-C**).

### Effect of raising RZT by 3°C on proteins, elements, and metabolites

**Figure 4:**
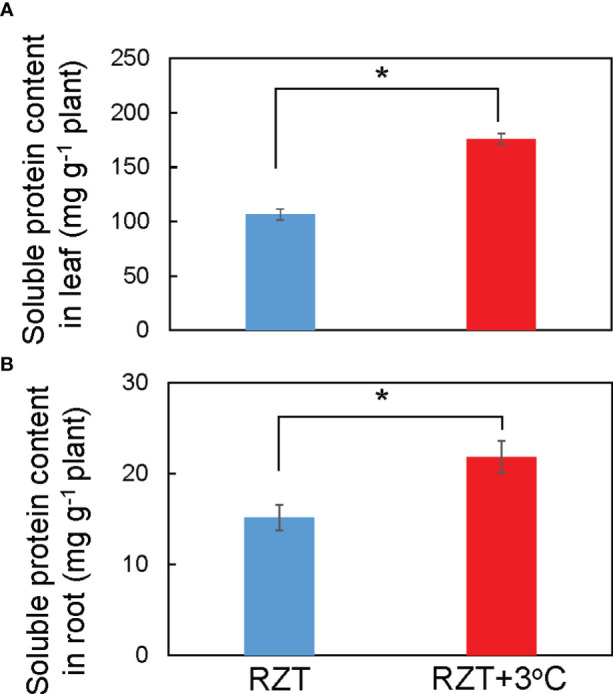
Soluble protein concentrations in the leaves **(A)** and roots **(B)** of ‘Red Fire’ red leaf lettuce grown in an unheated RZT and a +3°C RZT treatments at 22°C air temperature. Statistical differences between treatments at the same temperature by Tukey’s HSD test (5% level of significance). Bars are standard errors (n = 6-8). The mark * indicates significant differences between treatments at the same temperature.

Raising RZT significantly increased total soluble protein in the roots by 31% and leaves by 40% of plants grown at 22°C relative to its corresponding unheated root zone treatment (**Figures 4A, B**). Ionome and metabolome analyses were performed on plants grown at the air temperature of 22°C to further elucidate the mechanism by which raising the RZT by 3°C promoted plant growth and improved metabolites (ascorbic acid and chlorophyll).

**Figure 5:**
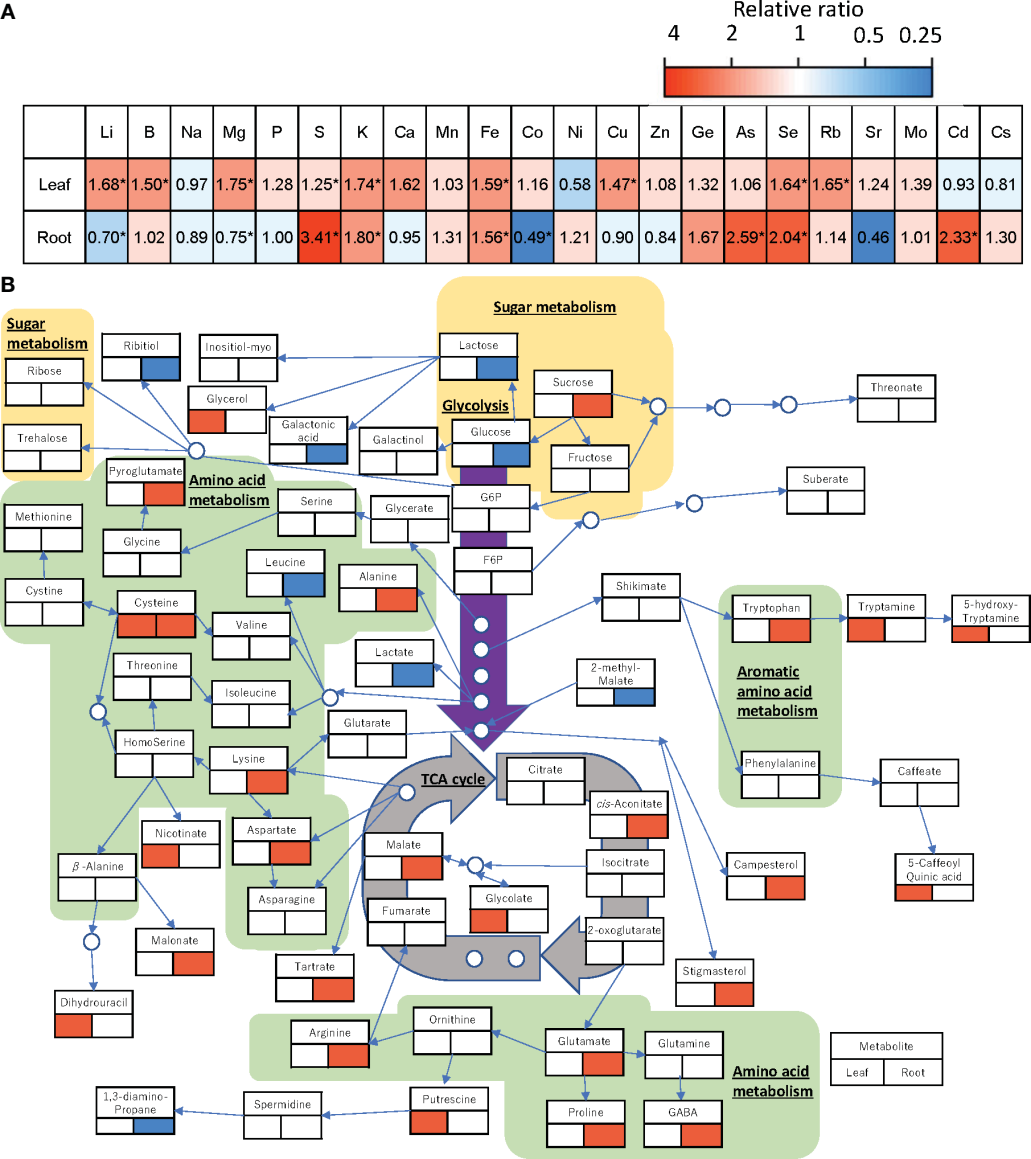
Changes in the relative amount of each element **(A)** and each metabolite **(B)** in the leaf and root of ‘Red Fire’ red leaf lettuce grown under heated RZT in the 22°C air temperature treatments. Significant increases are shown in red and decreases in blue by a Tukey’s HSD test (5% level of significance). The lower left of the metabolite names represents changes in metabolites in the leaves and the lower right represents changes in metabolites in the roots. * **(B)** can only be used for general interactions, and it is not a complete comprehensive diagram of all metabolomic interactions.

Analysis of ionome profiles showed that raising the RZT by 3°C affected various elements of the roots and shoots of plants grown at the air temperature of 22°C (**Figure 5A**). In the leaves, Li, B, K, Fe, Cu, Se, and Rb were significantly higher in the RZT+3°C treatments than the unheated RZT treatments, but in the roots, Li, Mg, and Ca were significantly lower, while S, K, Fe, As, Se, and Cd were significantly higher (**Figure 5A**).

Raising the RZT by 3°C also affected the metabolic profiles of plants grown at 22°C (**Figure 5B**). In the roots, it decreased glucose but increased sucrose and malate (**Figure 5B**), and it significantly increased 10 amino acids (alanine, arginine, aspartate, cysteine, GABA, lysine, proline, pyroglutamate, glutamate, and tryptophan) (**Figure 5B**). Although it had no significant impact on metabolic profiles, the leaves showed increased glycerol as a carbohydrate, and 5-caffeoylquinic acid (chlorogenic acid) (**Figure 5B**).

### Clustering elements and metabolites altered by raising the RZT by 3°C

**Figure 6:**
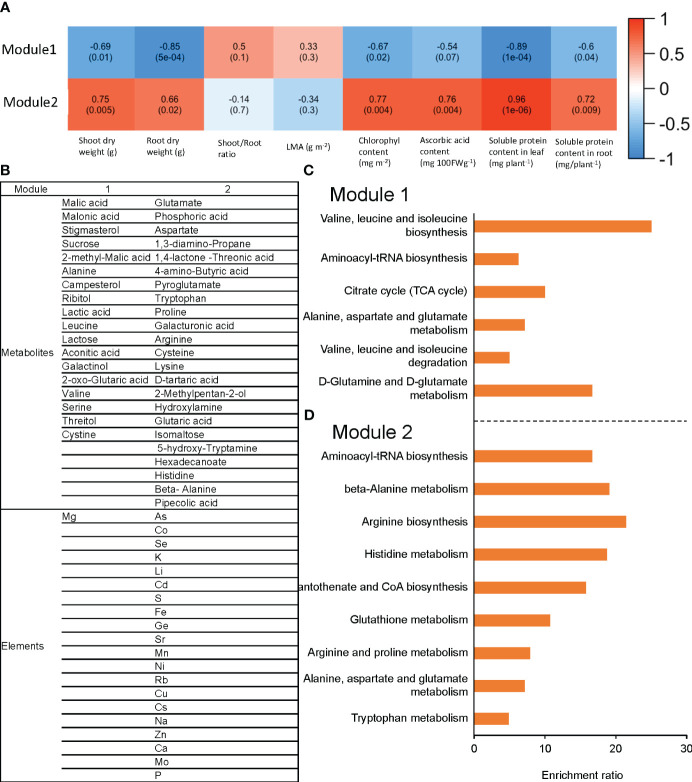
The correlation between each module and phenotype for ‘Red Fire’ red leaf lettuce is shown in **(A)**. This module consists of the measured elements and metabolites with significantly different concentrations with Tukey’s HSD test (5% level of significance) caused by raising the RZT in the 22°C air temperature treatments. The list of metabolites and elements for each module are shown in **(B)**; **(C)** shows metabolite enrichment analysis in Module 1; and **(D)** shows metabolite enrichment analysis in Module 2. WGCNA elements and metabolites were classified into two modules (Module 1 and Module 2), based on their correlation with shoot and root dry weights, chlorophyll, ascorbic acid, leaf soluble protein, and root soluble protein.

Raising the RZT by 3°C in plants grown at an air temperature of 22°C changed the metabolites and elements. The metabolites and elements were clustered by network analysis using WGCNA and classified into two modules (Module1 and Module2). Module1 elements and metabolites were positively correlated with shoot and root dry weights, chlorophyll, ascorbic acid, leaf soluble protein, and root soluble protein. The elements and metabolites of Module2 showed a negative correlation with all the variables that were found to be correlated with Module1 (**Figure 6A**).

Module1 contained 18 metabolites and 1 element, while Module2 contained 23 metabolites and 20 elements (**Figure 6B**). Enrichment analysis (Metabo4.0) was used to determine the metabolic pathways enriched for the metabolites in these modules. Module1 was significantly enriched with valine, leucine, and isoleucine biosynthesis, aminoacyl-tRNA biosynthesis, and citrate cycle (TCA cycle) (**Figure 6C**). In contrast, Module2 was significantly enriched with aminoacyl-tRNA biosynthesis, beta-Alanine metabolism, and arginine biosynthesis (**Figure 6D**).

### Principle component analysis

**Figure 7:**
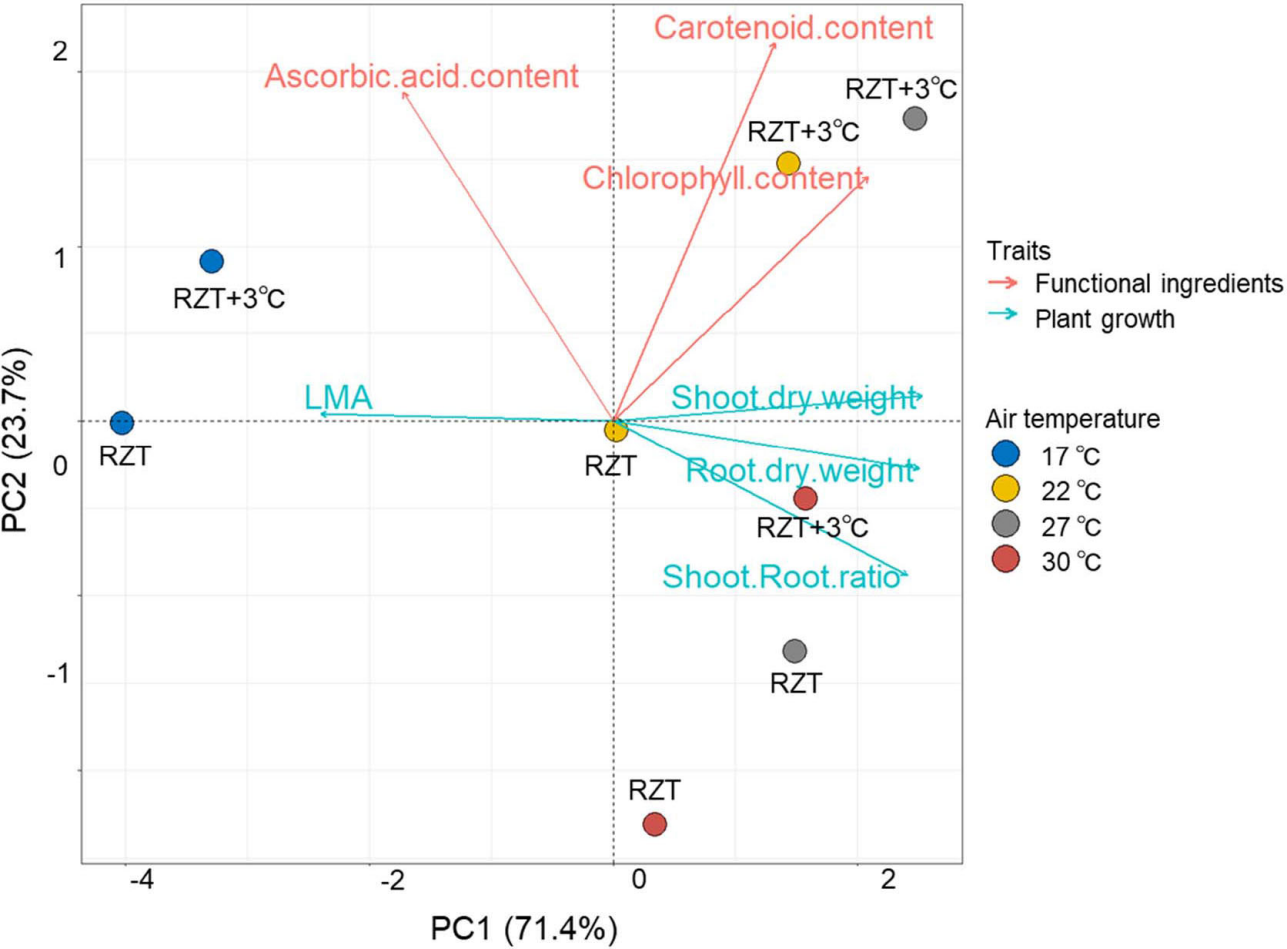
Principle component analysis (PCA) of the traits for ‘Red Fire’ red leaf lettuce grown at different temperatures. Arrows indicate the direction and strength of each trait’s contribution on the first two PCs.

Principle Component Analysis (PCA) was performed to identify the relationships of traits with plant growth and metabolites (**Figure 7**). The first two principal components, PC1 and PC2, accounted for 70.3% and 20.0% of the total variance, respectively. In PC1, shoot and root dry weights, shoot/root ratio, and chlorophyll concentrations showed positive values, whereas LMA showed negative values. Ascorbic acid and carotenoid concentrations showed negative values in PC2.

### Two-factor ANOVA analysis

Overall, the results suggest that the air temperature and RZT combined only significantly interacted with root dry weight (**Table 1**). They did not significantly affect ascorbic acid concentrations, shoot dry weight, shoot/root ratio, LMA, chlorophyll, and carotenoid concentrations (**Table 1**). The RZT alone had significant interactions with root and shoot dry weights, chlorophyll, carotenoid, and ascorbic acid (**Table 1**), while the air temperature alone significantly interacted with root and shoot dry weights, LMA, chlorophyll, carotenoid, and ascorbic acid (**Table 1**).

**Table 1:** Show differences in traits of plant growth and functional ingredients when grown at different air and root zone temperatures.

Air temp.	RZT	Root dry weight (g)		Shoot dry weight (g)		Shoot/root ratio		LMA (g m^-2^)		Chlorophyll (mg g^-1^ FW)		Carotenoid (mg g^-1^ FW)		Ascorbic acid (mg 100g^-1^ FW)	
17°C	+0°C	0.34e		0.109e		3.13c		1.67a		0.92b		0.230ab		9.88a	
	+3°C	0.42e		0.155e		2.80c		1.56ab		1.13ab		0.260ab		10.2a	
22°C	+0°C	1.37d		0.247d		5.59b		0.91c		1.35ab		0.273ab		5.38bcd	
	+3°C	1.99c		0.327bc		6.17ab		0.85c		1.72a		0.315ab		7.44ab	
27°C	+0°C	2.23bc		0.307bc		7.34a		0.68c		1.38ab		0.232ab		3.59d	
	+3°C	2.73a		0.394a		7.02a		0.83c		1.65a		0.354a		7.31acb	
30°C	+0°C	2.12c		0.294cd		7.32a		1.15bc		1.06ab		0.213b		2.58d	
	+3°C	2.47ab		0.361ab		6.96a		1.04c		1.42ab		0.278ab		3.92cd	
mean	17°C	0.38d		0.132d		2.96c		1.61a		1.04b		0.247a		10.0a	
	22°C	1.68c		0.287c		5.88b		0.88bc		1.53a		0.294a		6.30b	
	27°C	2.48a		0.350a		7.18a		0.76c		1.51a		0.281a		5.45b	
	30°C	2.29b		0.327b		7.14a		1.09b		1.27ab		0.252a		3.15c	
mean	+0°C	1.51a		0.239a		5.84a		1.10a		1.19a		0.238b		5.09b	
	+3°C	1.90b		0.309b		5.74a		1.07a		1.47b		0.298a		7.25a	
ANOVA	Air temp.	***		***		***		***		**		n.s.		***	
	RZT	***		***		n.s.		n.s.		**		**		***	
	Air temp. x RZT	***		n.s.		n.s.		n.s.		n.s.		n.s.		n.s.	

** and ***, significant at the 0.01 and 0.001 levels; n.s., not significant (n=4~7). Different letters indicate significant differences among treatments according to Tukey’s test (p < 0.05).

### Energy analysis of raising RZT by 3°C on lettuce

The energy consumption in kilowatt-hours (kWh) per 1 g fresh weight of fresh lettuce shoot biomass was 0.236, 0.037, 0.046, and 0.067 kWh for root zone heating at the 17, 22, 27, and 30°C treatments, respectively.

## Discussion

This study showed two key points. First, raising the RZT by 3°C relative to any of the four air temperatures enhanced total soluble protein, various elements, and amino acids in roots, resulting in improved plant growth and yields. Second, it increased the concentration of carotenoid and chlorophyll metabolites and ascorbic acids at most air temperatures, suggesting improved quality since these are often desirable compounds for consumers. Some undesirable element uptake was significantly increased in the roots with a RZT increase of 3°C, such as Cd and As. Still, they did not accumulate significantly in the leaf tissue, which is the frequently consumed part of the plant. PCA revealed that shoot and root dry weights had similar trends as chlorophyll concentrations but had a weak relationship with ascorbic acid and carotenoid concentrations. These results suggest that plant growth and metabolites could be improved simultaneously. In summary, raising the RZT 3°C above the air temperature results in enhanced total soluble protein, various elements, and amino acids in roots, improved plant growth, and yields relative to our unheated control treatments. Further studies would be needed to investigate the optimum RZT relative to the air temperature by investigating multiple RZT differences relative to air temperature.

### Raising RZT by 3°C increases the metabolite, soluble protein, amino acid, and elements of lettuce

In all temperature treatments, raising the RZT by 3°C increased chlorophyll, carotenoid, and ascorbic acid (**Figure 3**). Previous studies have suggested that increases in certain antioxidants generally occur under stressful conditions (Perrin and Gave, 1986; Smirnoff, 2000; Giannakourou and Taoukis, 2003; Hsu et al., 2013). Furthermore, the plants grown at an air temperature of 22°C and RZT+3°C had significantly increased concentrations of chlorogenic acid in their leaves (**Figure 5**). Chlorogenic acid is a polyphenol with antioxidant properties that may reduce oxidative damage in human cells (Khanam et al., 2012). Thus, raising the RZT increased chlorophyll, ascorbic acid, and chlorogenic acid, antioxidants that could bring added health benefits to the consumer.

Based on the analysis of metabolic profiles, raising the RZT by 3°C increased the concentrations of total soluble proteins (**Figure 4**) and various amino acids (**Figure 5B**) in root and leaf tissue. The lower soluble protein content in the unheated RZT treatment could be attributed to numerous factors. One possibility is that a lower RZT leads to the progressive degradation or retardation of proteins because more energy carriers are consumed to enhance adaptation to low temperatures, producing lipids, amino acids, and other molecules, in addition to promoting cell membrane fluidity and structural rearrangement (Maruyama et al., 2014; Wu et al., 2016). Another possibility is that cold temperatures also caused the destabilization of protein complexes (Thakur and Nayyar, 2013), while high RZT treatments have also been reported to lead to a decline in soluble protein as well since high RZT stress causes root protein to decrease. At the same time, sucrose metabolism is activated to store energy for root survival, resulting in the accumulation of sugars instead (Du and Tachibana, 1994). These responses would lead to increase in the concentrations of total soluble proteins by raising the RZT by 3°C, whereas decrease in the unheated RZT treatment.

Furthermore, nutrient uptake and the metabolism of amino acids in root and leaf tissue were enhanced at all four air temperatures, with the concentrations of aspartic acid and glutamine mainly increased (**Figure 5**), which aspartic acid and glutamic acid are the starting amino acids for the biosynthesis of many amino acids (Jander and Joshi, 2010; Walker and van der Donk, 2016; Appleton and Rosentrater, 2021). Arginine biosynthesis and β-alanine metabolism were also positively correlated with protein concentrations and plant growth parameters (**Figure 6**), as arginine metabolism is believed to promote plant growth (Kawade et al., 2020) and β-alanine is a precursor of CoA that is involved in producing fatty acids, which are also cellular building blocks (Parthasarathy et al., 2019). Overall, this increase in biochemical properties may have led to an increase in photosynthesis and, thus, in biomass. However, the biosynthesis of valine, leucine, and isoleucine showed a negative correlation with protein concentrations and plant growth parameters (**Figure 6**), and isoleucine decreased as the RZT was raised (**Figure 5**). As valine, leucine, and isoleucine are reported to increase in reaction to the stress response (Joshi et al., 2010), there is a trade-off between plant growth and defense (Huot et al., 2014) as growth–defense tradeoffs are considered to occur in plants due to resource restrictions, which demand prioritization towards either growth or defense.

Based on the analysis of ionome, the concentrations of various elements were found to be increased upon raising the RZT, with Mg, K, and Fe being the most significantly affected (**Figure 5A**). Interestingly, the observed increase in Mg levels aligns with the findings of other studies, which have shown that raising the RZT for wheat leads to an increase in Mg uptake in the first 30 days of growth (Huang and Grunes, 1992). Further studies on NH_4_ and K uptake have found reductions when RZT is reduced, which is also consistent with our results for K uptake (Shabala and Shabala, 2002). According to our research, there was a noticeable increase in S uptake, which aligns with the findings of studies on onions where the RZT was raised to 21°C (Coolong and Randle, 2006). Furthermore, we found a significant increase in B in leaf tissue, consistent with other research studies that concluded that B uptake decreases at lower RZTs (Ye et al., 2000). Another study with tomatoes found the optimal RZT for Cu, Mn, and K to be around 24°C, consistent with our results (Tindall et al., 1990).

Furthermore, in the leaves, Fe and Se were significantly greater with RZT+3°C than with RZT in plants grown at an air temperature of 22°C (**Figure 5**). It is worth noting that Fe is essential for human health (Abbaspour et al., 2014), and Se has antioxidant properties that contribute to the prevention of epidemics in humans (Xiao et al., 2021). These results are consistent with other studies of tomatoes that have found that raising root temperatures increased Fe and Mn uptakes (Riekels and Lingle, 1966). Raising the RZT significantly changed root and shoot elemental composition uptake.

### Raising RTZ by 3°C increases the value of lettuce

We examined whether raising the RZT by 3°C could be viable option in lettuce production. Raising the RZT by 3°C relative to any of the four air temperatures enhanced plant growth and yields (**Figure 2**). Energy consumption analysis proved that the 22 and 27°C air temperature treatments combined with raising the RZT were most beneficial. However, there are still challenges in implementing RZT heating controls in commercial plant factories. In plant factories, plants are typically grown in vertical, multi-stage rows, so it may be more expensive to implement heating controls in all the rows (Kozai, 2013). However, creating a more sustainable system might be possible if the waste heat from the LEDs could be used to raise the RZT for each cultivation rack. Furthermore, the cost of air conditioning should be considered as the heat generated by increasing the RZT may escape into the air, requiring more energy from the air conditioners.

This research could also be applied to greenhouses and open fields. In greenhouse environments, research similar to raising the RZT has been conducted on heating strawberry crowns to increase yields (Kawade et al., 2020). As hydroponics in greenhouses has become more common, raising the RZT by heating the culture medium to increase yields may be possible. Even with conventional field production practices, raising the RZT has been conducted by covering the soil with insulating mulch during winter (Deschamps and Agehara, 2019), which indicates that actively increasing the RZT could promote plant growth in field conditions.

## Conclusion

This study demonstrates that raising the RZT by 3°C increased nutrient uptake from the roots to the leaves, and in the roots, it increased various amino acids and total soluble proteins. Furthermore, plant growth and metabolites (carotenoids, ascorbic acids, and chlorophyll) were improved in a broad range of air temperatures. The results also indicate that in plant factories, raising the RZT may increase productivity and the value of lettuces grown. In the future, constructing a plant factory with RZT heating capabilities could be beneficial. Furthermore, looking into the nighttime versus daytime effects on plant growth and heating effects on the lettuce stem could be helpful.

## Data availability statement

The original contributions presented in the study are included in the article/**Supplementary Material**. Further inquiries can be directed to the corresponding author.

## Author contributions

SH: Data curation, Formal analysis, Methodology, Visualization, Writing – original draft, Writing – review & editing. CL: Data curation, Formal analysis, Methodology, Visualization, Writing – original draft, Writing – review & editing. WYu: Data curation, Visualization, Writing – review & editing. MU: Data curation, Investigation, Writing – review & editing. AY: Data curation, Investigation, Writing – review & editing. YO: Data curation, Methodology, Validation, Visualization, Writing – review & editing. MKu: Data curation, Investigation, Validation, Writing – original draft, Writing – review & editing. MKo: Data curation, Investigation, Writing – review & editing. TN: Data curation, Investigation, Writing – review & editing. IK: Data curation, Investigation, Writing – review & editing. SK: Data curation, Investigation, Writing – review & editing. WYa: Conceptualization, Data curation, Funding acquisition, Project administration, Supervision, Validation, Writing – original draft, Writing – review & editing.
