# Supplementary material for: Raising root zone temperature improves plant productivity and metabolites in hydroponic lettuce production
Source: Front Plant Sci. 2024 Apr 16;15:1352331. doi: 10.3389/fpls.2024.1352331 (PMC11058216; doi:10.3389/fpls.2024.1352331)
Supplement: Supplementary file 1 [file DataSheet_1.pdf]

Raising Root Zone Temperature Improves Plant Productivity and Metabolites in Hydroponic Lettuce Production

Sota Hayashi[1], Christopher P. Levine[1], Wakabayashi Yu[1], Mayumi Usui[2], Atsuyuki Yukawa[2], Yoshihiro Ohmori[1], Miyako Kusano[3,4,5], Makoto Kobayashi[5], Tomoko Nishizawa[5], Ikusaburo Kurimoto[6], Saneyuki Kawabata[1], Wataru Yamori[1*]

[1] Institute for Sustainable Agro-ecosystem Services, The University of Tokyo, Nishitokyo, Tokyo, Japan

[2] Plants Laboratory Inc., Tanashi, Nishitokyo, Japan

[3] Faculty of Life and Environmental Sciences, University of Tsukuba, 1-1-1 Tennodai, Tsukuba, Japan

[4] Tsukuba-Plant Innovation Research Center (T-PIRC), University of Tsukuba, 1-1-1 Tennodai, Tsukuba, Japan

[5] Riken Center for Sustainable Resource Science, Yokohama, Kanagawa, Japan

[6] National Institute of Technology, Kisarazu College, Kisarazu, Chiba, Japan

*** Correspondence:**
Wataru Yamori
yamori@g.ecc.u-tokyo.ac.jp

Keywords: Root zone temperature, metabolome, pigment, ionome, lettuce (*Lactuca sativa*), plant factory

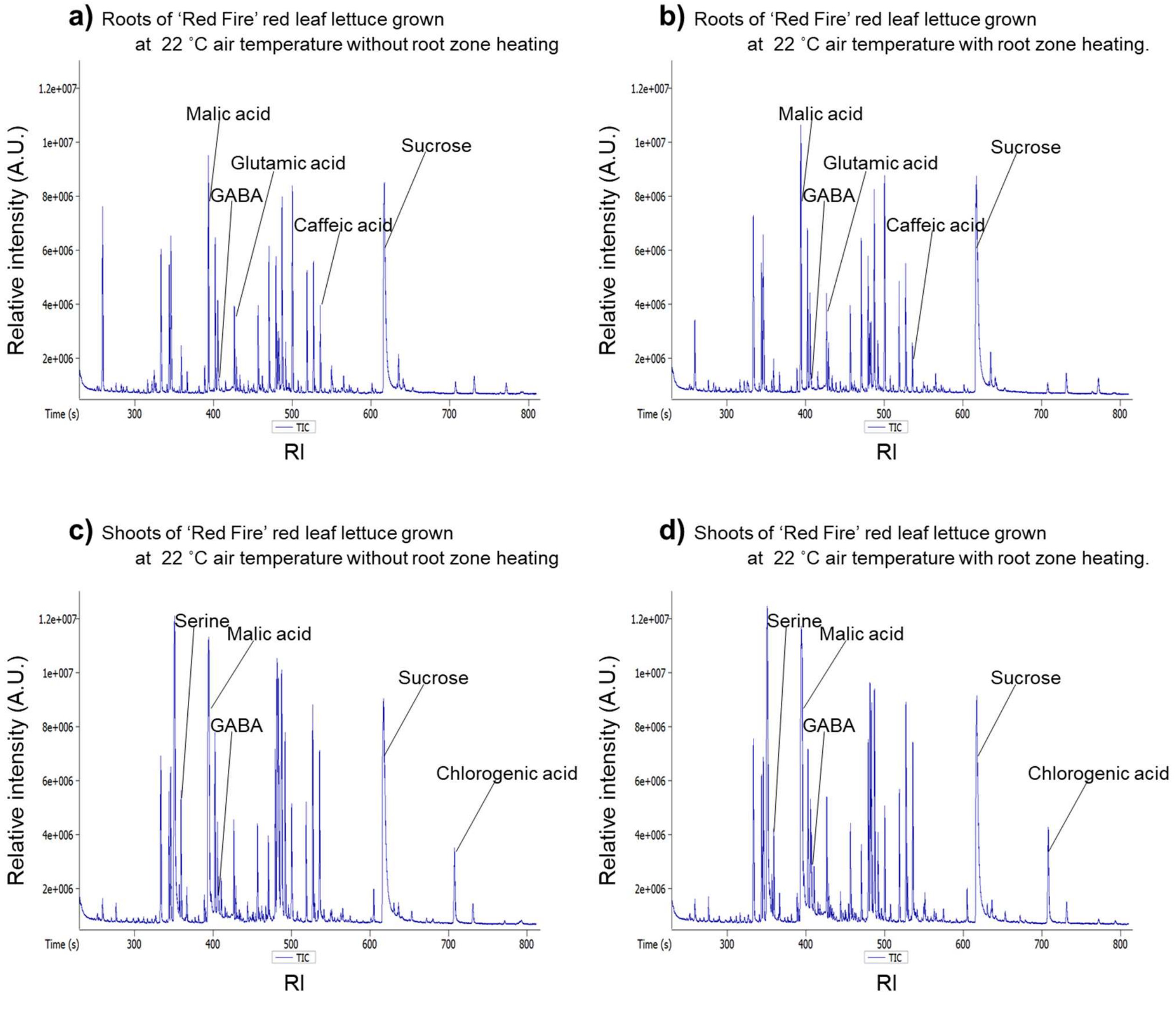

Figure S1. Representative total ion chromatograms (TICs) obtained by GC-MS analysis for (a) roots at 22 ˚C air temperature without root zone heating, (b) roots at 22 ˚C air temperature with root zone heating, (c) shoots at 22 ˚C air temperature without root zone heating, (d) shoots at 22 ˚C air temperature with root zone heating.
